# Conversations with God: How Are Religion and Spirituality Used to Make Sense of Forgiveness?

**DOI:** 10.1007/s11089-023-01081-z

**Published:** 2023-06-10

**Authors:** Anne Haikola

**Affiliations:** Department of Social Sciences, Social Psychology, University of Eastern Finland, Yliopistonranta 1, 70600 Kuopio, Finland

**Keywords:** Forgiveness, Transgression, Revenge, Religion, Spirituality, Narrative analysis

## Abstract

Forgiveness has a connection to religion and spirituality. Yet, little is known about how religious and spiritual people actually forgive. The present study investigated how religion and spirituality are used to make sense of forgiveness. The narratives of seven interviewees were chosen for close analysis of their experiences of forgiveness. McAdams’s life story interview method and narrative analysis were applied. Five themes were formulated: (1) forgiveness as Christian duty, (2) forgiveness as God’s miracle, (3) forgiveness through praying, (4) forgiveness through God’s sacrifice, and (5) forgiveness as God’s mercy. The findings indicate that God was important to the interviewees and supported their forgiveness process. Subthemes of revenge and justice suggest that sometimes forgiveness and revenge motives may be intertwined. Forgiveness was a divine process for the participants, and some felt that they would not have been able to forgive without God. Attributing forgiveness to God may serve the forgiveness process.

## Introduction

Offences are a fundamental part of human life and relationships. When one faces a transgression, there are roughly three options: avenge, ruminate and let it be, or forgive. For many people, the latter option seems to be the hardest one (Brüne et al., 2013; McGrath, 2015). Researchers have debated for decades the nature of forgiveness (Worthington, 2005). Many agree that forgiveness involves a prosocial change towards the transgressor (Witvliet, 2020, pp. 167–168) or, more precisely, a set of changes (McCullough, 2001). Forgiveness has been defined as a psychological process which transforms a person’s cognitions (Zheng et al., 2015), emotions (Kachadourian et al., 2004), physiology (Larsen et al., 2012), motives (McCullough et al., 2006), behaviour (Billingsley & Losin, 2017), and relationships (Strelan et al., 2017). Not everyone experiences all these changes similarly since forgiveness is rarely a static state (Abrahamson et al., 2012).

What usually makes forgiveness so difficult is the stress and emotional turmoil it causes, which one needs to manage somehow (Martinez-Diaz et al., 2021; Toussaint et al., 2020, p. 186). Indeed, unforgiveness has been defined as a stress reaction (Worthington & Scherer, 2004). Offence is a betrayal of trust (Strelan et al., 2017), which the victim interprets as a signal of disrespect (Miller, 2001). Transgressions are hurtful acts (Fincham et al., 2005), which break the need to belong (Baumeister & Leary, 1995) and violate the sense of self and meaning in life (Nezlek et al., 2012). Many experience the need for revenge or avoidance and diminishing friendliness towards the transgressor as a result of offence (McCullough et al., 2006). Considering an offender compassionately may be slightly cognitively demanding (Will et al., 2015; Witvliet et al., 2015). The offender’s post-transgression behaviour affects the likelihood of revenge and forgiveness (Martinez-Diaz et al., 2021). One may need time to move from unforgiveness to forgiveness (Williamson et al., 2014).

The literature encompasses different types of forgiveness. The types include forgiveness of others (Lee & Enright, 2019), forgiveness of self (Wohl & McLaughlin, 2014), forgiveness of situations such as a hurricane or illness (Thompson et al., 2005), group forgiveness (Enright et al., 2016), and divine forgiveness (Fincham, 2020), which means forgiveness by God. As the presented literature shows, forgiveness is a multidimensional process which affects people on many levels. In this study, I approach forgiveness in the context of religion and spirituality since these are connected. I explore how religion and spirituality are used to make sense of forgiveness.

## Forgiveness, religion, and spirituality

Definitions of religion and spirituality vary (Nynäs et al., 2022). Religion may be defined as an institutional and collective phenomenon which is also part of the individual’s identity (Stolz & Könemann, 2016, pp. 1–5). I understand spirituality as a large concept which includes experiential, existential and cognitive elements. It may or may not be linked to religious communities (la Cour & Hvidt, 2010; MacDonald, 2000). I approach these concepts in the religious context of Finland, where 65.1% of the population belong to the Evangelical Lutheran Church of Finland (ELCF Statistics, n.d.) and 1.03% to the Orthodox Church of Finland (OCF Statistics, n.d.). Although not all Finns are members of religious and spiritual communities, the majority are, and religions also affect nonbelievers (Exline, 2020, p. 121).

Forgiveness is part of Christian belief and practice (Schnabl Schweitzer, 2010) and is meaningful for religious individuals and communities (McCullough et al., 2005). Christianity has likely made forgiveness a central phenomenon (Murphy, 2003, pp. 87–88), although its roots can be seen in ancient Greece (Griswold & Konstan, 2012). The idea of forgiveness and reconciliation is crystal clear in Christianity: all people are saved through God’s work of salvation (Smedes, 1998, pp. 352–353). It is stated that forgiveness and reconciliation cannot be separated in Christianity – they are deeply intertwined (Bash, 2014). God’s forgiveness is love, and it should manifest on the individual and communal level, at best even beyond that (Marty, 1998, p. 11). Although God’s forgiveness is perfect and humans cannot reach the same divine level, they can still try to pursue that ideal. As individuals receive God’s forgiveness, they in turn seek to forgive other people (Bash, 2007, p. 94). It is assumed that Jesus’ teachings have had a remarkable impact on the advancement of forgiveness in Western culture and ethics. Although forgiveness may be understood as a religious and spiritual construct, it is also a secular and universal virtue (Bash, 2013).

Researchers have found that forgiveness is related to religion and spirituality (Choe et al., 2020) and that religious individuals have positive attitudes towards forgiveness (Matuszewski & Moroń, 2022). Religious people have been shown to possess higher levels of forgiveness and seeking forgiveness than nonreligious people (Toussaint & Williams, 2008). Escher (2013) found that believing in God’s forgiving promotes forgiveness of others and oneself. Further studies by Park (2005, 2007) show that religion may influence coping with life stress and can support well-being. Religion may promote resilience when people face hardship (Pirutinsky et al., 2020), people may turn to God during difficult times (Park, 2021, p. 234), and connecting with God through praying can bring comfort, forgiveness, and improved mental health (Black et al., 2015; Dein & Cook, 2015; Dein & Littlewood, 2007).

Nevertheless, it has not been very clear whether religious and spiritual people actually forgive more often than nonreligious people. Forgiveness concerns social desirability (Matuszewski & Moroń, 2022), and religion is correlated with social desirability (Trimble, 1997). Although religious and spiritual people may appreciate forgiveness more than nonreligious people, they may not forgive more often (Choe et al., 2020; Rhoades et al., 2007). Religious organizations may make it hard for their members to be unforgiving, and valuing forgiveness may modify religious beliefs (Rhoades et al., 2007). There are also methodological problems related to the connection between religion and forgiveness (Tsang et al., 2005). Therefore, the connection between forgiveness and religion or spirituality may not be unambiguous.

In the present study, I take a narrative approach to forgiveness and ask: How are religion and spirituality used to make sense of forgiveness? More specifically, I pay attention to the ways participants’ accounts of forgiveness relate to their accounts of God. In this article, my aim is to contribute to the understanding of forgiveness as a process of relationship with God.

## Method

### Interviewing participants

Altogether, 23 participants were recruited to the study. Participants were recruited via an advertisement on public social media channels and through Finland’s two national churches: the Evangelical Lutheran Church of Finland and the Orthodox Church of Finland. The purpose was to find adults who had forgiven something during their lives. McAdams’s life story interview was applied, which is a semistructured thematic interview method (McAdams, 1985). The interview included a writing task; participants were asked to write a story or several paragraphs about their forgiveness process. The interview contained six themes: life situation, values, and worldviews; transgression; forgiveness process; relationship with the offender; meaning of forgiveness; and wishes for forgiveness regarding the future. I did not ask directly whether participants were members of religious or spiritual communities, but some mentioned these.

For this article, the focus is on the narratives of seven participants who clearly described religion and spirituality as part of their forgiveness narrative. Since the interviews were held during the COVID-19 pandemic, participants could choose the format of the interview; one participant wanted to answer only by writing, three interviews were performed face to face in eastern Finland, and three were conducted via a secured video connection as requested by participants. The six participants’ interviews took a total of 530 min. The average interview length was 88 min; the longest was 135 min and the shortest 60 min. Interviews were recorded and transcribed with permission. I did not ask participants to state their gender identity. Not all of them shared their age, but those who did ranged from 33 to 62. Participants had experienced transgressions related to abusive parents, the other parent’s inadequacy to give protection against an abusive (step)parent, being left by a partner, being accused of a false statement, and unpaid debt. In the results section, participants are referred to with codes (e.g., P1 stands for Participant 1).

## Analysis procedure

Thematic narrative analysis offered a suitable approach to the material (Riessman, 2008, pp. 53–76). I started to familiarize myself with the material by reading it several times and then by highlighting and making notes in the margins. I approached participants’ interviews separately as unique stories but also marked what they had in common, as well as noting contradictions and different voices. After that, I transferred the material to the ATLAS.ti software and created codes based on my highlighting and notes. Codes, organized by themes, consisted of one or several sentences depending on the breadth of participants’ accounts. Next, I began writing the analysis based on the coding. The results section presents the main themes that were formed in the data-driven analysis process, which focused on spirituality, religion, Christian tenets, and God within these accounts of forgiveness.

## Results

The selected participants constructed religion and spirituality in the forgiveness process in multiple ways. Generally, they spoke about the effects of forgiving somebody or themselves or not forgiving in the context of religion and spirituality. All seven considered forgiveness a value that one should follow in life. Despite this willingness to forgive, they recognized it was not always easy. I identified five ways of talking about forgiveness in relation to God. All participants talked about God in the context of the Christian religion. Also, two participants referred to God more broadly as an unnamed and unknown supernatural force.

### Forgiveness as Christian duty

Nearly all participants spoke about the importance of God, Jesus, and the Bible in their lives. In this sense, they narrated forgiveness as a wise guideline they should follow in their lives, because God encourages God’s followers to forgive. Participants understood that they should forgive other people and themselves because their religion demands it and it is therefore the right thing to do. This duty was described as both normative and a voluntary moral choice. The speaking tone was mostly positive, and participants described God as knowing the importance of forgiveness, even when people do not always understand it. P2 described how you can find unforgiveness from the deadly sin of hate:

Hate and bitterness, those are always like . . . destroying powers. You can see them in the world, that they are like horribly. . . And of course they affect also on inner level, they destroy inner well-being. . . . Actually, when you think about the seven deadly sins, there is hate. I guess it means the very unforgiveness, it is the opposite of forgiveness. (P2)

This participant described how hate and bitterness are a destructive force, both interpersonally and intrapersonally. They reasoned that hate actually means unforgiveness so one should avoid that annihilating force. Their narrative contained the idea that unforgiveness is harmful both for individual well-being and in a global perspective, where unforgiveness causes damage in conflicts, arguments, and wars. Unforgiveness destroys the individual, and the suffering expands to other people too. Unforgiveness is meaningful in a context where deadly sins are interpreted as ruining one’s relationship with God and turning people away from God. If unforgiveness is the Antichrist, forgiveness is God.

At the same time, almost all participants described difficulty doing this Christian duty, even though they wanted to and believed it was right. They knew that they would be forgiven only if they forgave others, but this insight did not make the process easy. Participants made references to some transgressions which they had not forgiven or to unresolved cases. One even expressed irritation at this external and fundamental order that religious people should obey no matter what (P6). P1 considered forgiveness a task:

[Forgiveness] is a will and a need. . . . And then it excellently becomes concrete via fasting, confession, and the Sacrament of Penance. It is work to be done. You need self-reflection, solitude, honesty and interaction in order to do that. . . . Some part inside myself or whatever God or higher power or deeper insight and perspective there is. I think it’s conscious. Then you need to choose it and really feel that have you forgiven, can you do it and what could help you. . . . I still don’t know if I understand it. The more I think about [forgiveness], the more it transforms into something wondrous. I realized that like love, it is also a huge power which goes beyond understanding. Kind of the same as forgiveness. (P1)

This participant described how forgiveness becomes a tangible task through fasting and penance. At the same time, they described forgiveness as a wonderful phenomenon which is hard to comprehend, like love. This quote contains the idea that in order to forgive you should find inside yourself some kind of understanding, spend time alone, and interact with God or a higher power to enable you to accomplish something so difficult for human beings. Forgiveness was described as a concrete and palpable but also as a numinous phenomenon. This meaning mixed material and immaterial contexts to construct forgiveness as multidimensional.

### Forgiveness as God’s miracle

Some participants narrated forgiveness as a miraculous and wonderful phenomenon. Forgiveness was constructed as numinous and God’s incredible work, which reaches beyond everyday life. Forgiveness as a miracle was described as extraordinary and unbelievable yet real and concrete, and God was the powerful agent who made it happen. This theme contained narratives in which ultimate forgiveness happened suddenly and unexpectedly, like a natural phenomenon, although a process and work preceded it. One participant described God’s inexplicable work in themself and their father:

It was God’s work in me that I and my father had the [forgiveness] moment. And also that my father asked me for forgiveness. So, I see that it has been God’s miracle which God made in our hearts. I pray that God will continue God’s work in us so that we can get past those difficult years. (P6)

This participant conceptualized forgiveness as God’s guidance or miracle that goes beyond human understanding. In this sense, God’s power through the participant and their father brought about forgiveness and reconciliation. This participant wished that God’s work would continue so that they could get through the obstacles and their relationship with their father would become closer. This may not be in human hands; God’s help is needed to ensure forgiveness. For P7 the forgiveness process was directly linked to loss and bereavement. Yet, they expressed gratitude:

It was an unforgettable moment when I stood at my friend’s coffin. I felt grateful for remembering our shared moments together when my friend was alive. I was particularly pleased by the fact that I had met my friend accidentally about six months ago and then I forgave my friend’s wrongdoing voluntarily. My friend’s death was a total surprise to me. I thought that the higher power was present again, leading me to face my friend and giving me the opportunity to forgive face to face. I pray every day for guidance and protection in my life. I want to believe it, since it makes life more meaningful. (P7)

This participant expressed that forgiving the transgressor face to face was important. They considered it God’s guidance, which had been present in their life many times. For a Christian, acting on Christian values might be rewarding and feel right but might also involve some kind of normativity. After the interview, the participant wanted to ask me my personal thoughts about their experiences. Sometimes people want to feel that they are good when they forgive, and they may wish to receive affirmation from others.

### Forgiveness through praying

Some participants asked for God’s help in their forgiveness process and prayed for the power to forgive. Praying formed a connection with God and supported their forgiveness process. These participants gained insight and realization via praying, and it gave them a new perspective. This larger perspective and deeper understanding enabled them to forgive the transgressor. Their connection with God through praying was described as warm, compassionate, and close. It seemed that this connection was pivotal and fundamental to the process for participants. P5 experienced a breakthrough moment in forgiveness during praying:

A picture where Jesus was on a cross appeared in my mind immediately [during praying]. And then I heard the sentence: “I gave everything, and I was just abandoned.” That sank very deep in me. Suddenly I realized that God understands my pain. And so it was. It wasn’t like there was anything minimizing or anything like that at all. It was only full understanding and like compassion to me. But then I also realized that God has experienced that pain more deeply than me. That my pain is actually pretty small. That God has experienced the whole world’s [pain]. Like God came here to meet God’s people and then they crucified God. That contrast was like all that [unforgiveness and pain] just melted away from me. In that very moment I knew that I wouldn’t be holding any grudge against my transgressor anymore after this. (P5)

Feeling seen by God, validation of pain, and God’s compassion helped this participant to forgive during praying, even though earlier they had confessed to God that they could not forgive. But connection with God via praying and the deep sense that God saw their pain allowed this participant to let go. This forgiveness narrative has similarities to the therapeutic or parental bond. When God held the participant’s difficult feelings, healing could start, and the participant was finally able to forgive. What helped the participant was the sense that God had suffered much more, had felt and carried all the world’s pain, and had been betrayed and abandoned too. The participant’s own pain diminished and disappeared as a consequence of this broader perspective.

Sometimes God works through many people to bring about forgiveness. P6 narrated how friends helped:

There were a lot of people praying for that [forgiveness] moment. I had sent a message to my 40 Christian friends and asked them, “Could you pray? I’m seeing my father today and I wish we could have a moment full of mercy and love.” And then it was real. It was amazing. (P6)

This participant described asking friends to support them before they met with their abusive father, whom they had decided to forgive face to face even though it felt terrifying. To their surprise, their father asked for forgiveness when they met. The participant described how they were sure that God had made forgiveness possible. In this narrative, the friends’ prayers was a divine force which enabled reconciliation, forgiveness, love, and mercy. The forgiveness happened in cooperation with God’s work. In this narrative, forgiveness is not only individual but also collective and divine.

## Forgiveness through God’s sacrifice

Religious images, insights, visions, the Holy Spirit, God, and the Bible helped nearly all participants to forgive and were fundamental to their forgiveness narratives. Jesus dying on the cross for people’s sins was understood as an example of perfect forgiveness of everyone, including themselves. Unconditional forgiveness was described in a compassionate and mindful tone. This perspective supported forgiveness for P5 and P6, who understood the crucifixion of Jesus as meaningful through their forgiveness process. P1 talked about the significance of Jesus’ teachings:

There is inevitably spiritual vocabulary in the context [of forgiveness]. Even though you wouldn’t necessarily think that way. . . . Like in the church and this idea of dying on a cross. There is a lot of spiritual pictures and vocabulary about forgiveness in the Christian world. . . . When you come to think of it, there is a lot of good content about forgiving in Jesus’ teachings. I think it’s good that there is that dying on a cross in God’s teachings. (P1)

This participant said it is evident that forgiveness involves a spiritual realm and is spiritual in essence. This participant made a connection between forgiveness and the crucifixion of Jesus. In this sense, Jesus was a pioneer of forgiveness, which still affects the lives of Christians and probably also of non-Christians. The crucifixion of Jesus may be seen as an example of unconditional forgiveness, which might be perfection and a divine form of forgiveness. This participant considered the crucifixion a good lesson. The next phrase is an example of forgiveness through the Holy Spirit:

I said that in the name of Jesus Christ I will forgive my father for this. He doesn’t owe me this thing. Instead, he is like washed pure in the blood of Jesus. And inside me, where my feelings don’t accept this or my feelings are against it, there is Jesus’ blood which covers the sin. So, I was able to forgive my father. . . . I had a chance to forgive through the Holy Spirit. (P6)

This forgiveness happened in an interaction with the higher power, and the participant described how part of them resisted and another part desired to actualize forgiveness. Feelings and forgiveness were in conflict, and the crucial factor for forgiveness was Jesus, whose blood mitigated their father’s sin. It is notable that the participant’s expressions about Jesus’ blood washing away the sins are often used in one of the Finnish revival movements, the Laestadian movement. The participant may have been involved in this movement, even though they did not describe much about their religious background. They described the Holy Spirit as a mighty force above humans which enabled the tough act of forgiveness. This account included a meaning of forgiveness as an embodied ritual-like act which one performs with God.

### Forgiveness as God’s mercy

Three participants’ (P3, P4, P6) expressions of forgiveness were sometimes indirectly connected to subthemes of justice and revenge. Their narrations contained the meanings that it is important to forgive but that transgressors need to suffer for what they did. They described a pressing and haunting need to make things right and said that transgressors should be accountable for their actions. These narratives contained a normative, moral, and/or punitive tone. The avenger was conceptualized either as God or the law of karma, which was understood as spiritual in nature.

P6 had a difficult childhood because of abusive parents. Their forgiveness narration included the idea of getting even:

I said to my father, kind of pointed to him what he owes me. And at the same time when I read aloud from my letter what my father did to me, I said in every paragraph that in the name of Jesus Christ I will forgive you. He has paid for this too. (P6)

Debt may be interpreted as something that the transgressor needs to pay back or make amends for to the victim. Therefore, debt is part of revenge because the aim of revenge is to make things even. The subject of the last sentence was not defined, but it may be assumed that it is Jesus, who died for people’s sins. Because of Jesus’ sacrifice, the participant’s father’s debt has been paid as well. The mediator of revenge or the instrument of atonement was Jesus. What helped the participant to forgive was God telling them that they were God’s precious child. But throughout the participant’s childhood, their parents abused them, so this participant did not feel precious and dear. When they were able to process trauma and sensed God’s love on a personal level, this participant released their biological parents from debt via Jesus and forgave their parents.

P4 described how one cannot move on without forgiveness and how this message is from the Bible:

Forgiveness is quite a big message in the Bible. You can’t move on in life without it. Yes, it is pretty evident in the Bible. And also that God is the one who punishes at the end. A human’s only task and work is to forgive. Indeed, it is liberating. I think it is a strong teaching from religion. Who knows where I would be at this point [in my forgiveness process] without it. I don’t know, maybe not. (P4)

For this participant, the human being’s part is to forgive and God is the one who judges and punishes. They expressed how it felt liberating to abandon judgement and leave it to God, who would take care of it. They thought that they may not have been able to forgive without this idea. This account contained the meaning that there is fundamental order: God is the judge and the human a forgiver. Both fulfil their duties and work on different sides of the process. In this sense, forgiveness is not possible without God – divine forces are needed to actualize forgiveness and make it real for human beings. It might be that leaving judgement and justice to God may make room for an individual to concentrate on forgiveness and make the process easier. Also, justice does not always take place in the material world, so it might lessen the burden to consider its actualization in the immaterial world.

Some participants understood forgiveness as important to God but recognized that they were weak, wounded, angry, carnal, and limited human beings. They expressed that God knows better what is good and nourishing for people so Christians should do as God wishes. Some quoted from the Gospel of Matthew that if you forgive other people, God will forgive you too, but if you don’t, God will not forgive you either. Not all thought of this principle as an order; some considered it God’s love for people. But this idea of God as a judge partially helped one participant to forgive because they had experienced that God had made amends for their having abusive parents (P6). For this participant, God paid their parents’ debt by giving the participant wonderful things later in life and by loving them.

To summarize, God was present in participants’ forgiveness processes on a pivotal level. They asked for God’s help to forgive, and God answered that call by making forgiveness possible.

## Discussion

This study aimed to explore how participants used religion and spirituality to make sense of forgiveness after they faced transgression. God was important for the participants in their forgiveness processes. God supported their psychic processes and was pivotal to them. Participants made sense of religion and spirituality in the forgiveness process in multiple ways.

The theme of forgiveness as Christian duty included accounts of forgiveness traced to teachings from the Bible and Christianity. Participants considered it important to forgive because God wishes people to forgive. They described the difficulty of feeling that they could not forgive, even though they should be able to as Christians. Participants mentioned some transgressions which they had not forgiven. This is understandable since human beings are imperfect, have a limited capacity for forgiveness, and cannot forgive as lavishly as God does (Bash, 2007, p. 94). Forgiving has been found to cause stress if it is done only because one’s religion demands it (Cox et al., 2012). This study found minor indications of this, but mostly this theme had a positive tone. Participants described the Bible as a soothing support to rely on in the future when facing hard transgressions. In Christianity, the Bible is a way to have a connection with God, and participants found it to be a divine help in the forgiveness process. Earlier research has found that forgiveness has a self-soothing effect (Sandage & Jankowski, 2010), which supports the current findings. Individuals see God in relational terms (Wilt et al., 2021). Further, people perceive God and Jesus differently; Jesus may be seen as warmer and less fierce than God, although people might gain more from God than from Jesus (Cummings et al., 2017). These aspects might partially explain why God was constructed differently in the present study.

The theme of forgiveness as God’s miracle characterized forgiveness as God’s wonderful and inexplicable work. Forgiveness was described as God’s miracle which goes beyond human understanding. Forgiveness after suffering and pain may be interpreted as amazing and divine. God may be understood as the one who puts forgiveness into action in the material world. Harwood et al. (2022) found that forgiveness is one form of divine grace, which some participants seemed to experience. Forgiveness may be understood as God’s love in Christian religion (Marty, 1998, p. 11), and participants narrated forgiveness in the context of God’s love, mercy, and compassion. God’s work in forgiveness was constructed as tremendous and powerful, similar to the understanding in Christianity that God’s ability to manifest and transform forgiveness is almighty (Bash, 2007, pp. 94–95). Hyde and Joseph (2022) found that God’s grace aids people in forgiving others, as in the current study. People can make attributions to God when negative happenings end positively and frame God as a rescuer (Ray et al., 2015). However, it is common for religious people to formulate divine meanings in their lives (Pargament & Mahoney, 2005).

The theme of forgiveness through praying included accounts of help from God to forgive through praying. Some participants felt that they would not be able to forgive without God. Divine forces were needed to forgive, and praying helped in that process. For some participants, praying seemed to create an emotional bond with God, which enabled forgiveness. Individuals use distinct ways to have a connection with God (Cummings et al., 2017). It has been found that praying can facilitate forgiveness (Vasiliauskas & McMinn, 2013), and religion may foster a sense that God cares (McAdams & Dunlop, 2022, p. 311). Further studies have shown that connecting with God through praying may promote forgiveness, consolation, and better mental health (Black et al., 2015; Dein & Cook, 2015; Dein & Littlewood, 2007). People often pray for insight, wisdom, salvation, motivation (Plante, 2021, p. 261), and to have a dialogue with God (Gerundt et al., 2022), as this study’s participants did.

The theme of forgiveness through God’s sacrifice contained the meaning of perfect and unconditional forgiveness via Jesus. The crucifixion was constructed as a positive lesson from the Bible. This helped some participants to forgive and gave them the sense that they were forgiven too, like everyone else. When one thinks that all people are forgiven, it might make the forgiveness process easier. It has been confirmed that feeling forgiven by God lessens the need to witness acts of repentance from transgressors (Krause & Ellison, 2003), which was also demonstrated in the current study. It is understood in Christianity that all people are saved through God’s work of salvation (Smedes, 1998, pp. 352–353), and participants seemed to comprehend this idea. Participants’ narrations of Jesus highlight Jesus’ impact on the theological and cultural understanding of the essence of forgiveness (Bash, 2013). This model is nowadays called “the Christian ideal of forgiveness,” which means perfect and unconditional forgiveness without feelings of anger or vengeance (Bash, 2007, pp. 92–93).

The theme of forgiveness as God’s mercy included accounts of justice and revenge. It seemed that for some participants the idea that God would judge transgressors supported their forgiveness processes. Not all considered God a menacing judge, and some did not think that God forced people to forgive. Instead, some understood it as an act of love. The present findings indicate that sometimes forgiveness and revenge may be intertwined. Leaving judgement and justice to God seemed to serve participants’ forgiveness processes. Since justice does not always happen in the material world, it might be liberating to consider that God takes care of it. The transgressor will be punished, and the victim will not be the only one who has suffered. Religious understandings of forgiveness vary (Murphy, 2003, p. 88), and different religious meaning systems might encourage religious people either to forgive or to take revenge (Tsang et al., 2005). Indeed, researchers have started to realize that forgiveness motives may not always be purely benevolent; sometimes forgiveness may be a chance to manifest oneself as morally superior to the transgressor and take revenge for the offence (Gollwitzer & Okimoto, 2021; Wenzel & Okimoto, 2012). Consequently, there are many shades of forgiveness, and the process is likely to differ depending on whether forgiveness is considered as mercy or revenge. Future studies could investigate how people negotiate these different sides of forgiveness and what kind of narratives individuals create when they justify forgiveness as revenge.

Although some participants made statements about God as a punishing judge and used a punitive tone when they described God as an avenger, it seemed that this view did not harm them or their relationship with God. For some, it actually promoted forgiveness. There were no direct mentions of anger toward God in participants’ statements. Previous research has shown that people may be angry with God for the misfortune in their lives (Rudolfsson & Tidefors, 2014). Thus, this emotion may be stigmatized and people might not be willing to share it (Exline, 2020). Seeing God as cruel has also been connected to struggling with the idea of God (Exline et al., 2015).

God as an avenger was also demonstrated by Nyarko and Punamäki (2017) in their interviews with young adult survivors of war. The interviewees stated one should rely on God to get justice. People may perceive God as the supreme moral agent (Gray & Wegner, 2010). As in this study, it seems that attributing responsibility to God may support one’s forgiveness process. It has been confirmed that attributing responsibility is skewed; individuals often view their own behaviour as resulting from external factors (Morton, 2012) but others’ behaviour as resulting from internal factors (Kubota et al., 2014). Pargament et al. (1988) have defined three coping styles which religious people use: collaborative, deferring, and self-directing. Participants in the current study seemed to use collaborative and deferring styles when they took responsibility in the forgiveness process, shared the responsibility with God, or attributed the responsibility to God. Further, Pargament et al. (1998) have stated that people use religious forgiveness as a positive coping method, as in the current study.

Investigation of forgiving or not forgiving real life offences is relevant, and the present study contributes to the field. There have been relatively few studies exploring the whole process from transgression to forgiveness using in-depth interviews. Some researchers have explored factors influencing forgiveness and unforgiveness processes (Akhtar et al., 2017), children’s and adolescents’ narrative accounts of forgiveness and unforgiveness (Wainryb et al., 2020), and forgiveness as part of couples’ reconciliation process after infidelity (Abrahamson et al., 2012). This study shows how religious and spiritual individuals’ relationship and potential struggle with God affects their forgiveness process, which is important for helping them through that process.

This study adds to the literature the finding that having a religious or spiritual worldview may not always guarantee forgiveness since participants stated that they had not forgiven every transgression they had faced. Future studies could explore the conditions in which religious and spiritual people may forgive or not forgive. The findings may be used in health care, pastoral care, and clinical and counselling settings. Clinicians and pastors could support individuals’ relationship with God when they are facing transgressions. This might help people to eventually forgive. Maybe churches and other religious communities could arrange prayer events to foster forgiveness. It is important that clinicians understand the human and somewhat inherent desire for revenge and support people to find more adaptive coping strategies for dealing with transgressions.

Moreover, clinicians and pastors could explore individuals’ motives to forgive since these might be benevolent or less benevolent. It is also important to be aware of the normativity of forgiveness in religious communities and its possibly detrimental effect on individuals’ mental health. This forgiveness imperative may set limits on how one can behave and relate to people in the community. Care practices should take account different processes of forgiveness. Not every process of forgiveness is the same. Some people may experience forgiveness as a mandatory act and some as mercy. Practitioners should be conscious of these abundant shades of forgiveness and recognize variation and levels in the processes.

## Limitations

This study is not without limitations. It was impossible to confirm the participants’ narrated situations; people may narrate their experienced transgressions with bias (Dorn et al., 2014; Pronin, 2008). However, objective truth was not the goal of narrative study. Rather, the goal was to explore the truth of experience (Riessman, 1993). Forgiveness research has been criticized for mainly relying only on one method: asking people first to recall a particular offence and then to complete a self-report regarding that transgression (Dorn et al., 2014). A multi-method strategy – by talking and writing – was used to do this here. Forgiveness research also has a problematic tendency to focus on people’s attitudes and thoughts related to forgiveness. This may not be enough since reasoning is biased (Kunda, 1990), people do not necessarily know themselves as well as they think (Wilson & Dunn, 2004), individuals do not always behave according to their values (Chrystal et al., 2019), and people try to present themselves in a desirable light (Goffman, 1959). Therefore, behavioural measures of forgiveness are required (Tsang et al., 2005).

The number of participants in this study was relatively small, and the sample is not representative. However, the strength of narrative analysis is that it enables profound knowledge about human life and individual experiences (Riessman, 1993, p. 70), which forgiving certainly is. The researcher’s gender might have affected the interviews since women may be perceived stereotypically as empathetic and emotional. It is possible that participants were aware that the researcher was a licensed psychologist, which may have affected how they formulated their narratives. Participants might have been more reflective and highlighted the psychological side of forgiveness. Future studies could investigate forgiveness as an emotional and embodied process since precise knowledge related to this is scarce. Further, it is important to better comprehend similarities and differences between religious, spiritual, and nonreligious people. Although forgiveness is a universal phenomenon, it may have various meanings and manifestations depending on the worldview and the culture in which individual lives are lived; this makes it relational in nature (Bash, 2013, 2014). The forgiveness process is individual, and for some it may be divine.

## Conclusions

The findings of the study indicate that religion and spirituality are used to make sense of forgiveness in multiple ways. God was important to the participants and supported their forgiveness process. The participants’ accounts suggest that forgiveness may be a divine process and that one may not be able to forgive without God. Usually, revenge and forgiveness are considered as separate processes, but in the current study there were indications that forgiveness and revenge were intertwined. What might link them is a sense of justice. Participants seemed to attribute a role to God in the forgiveness process. Externalizing forgiveness to God appeared to help them when they were navigating from unforgiveness to forgiveness.
